# *N*-(5-Methyl­sulfanyl-1,3,4-thia­diazol-2-yl)acetamide

**DOI:** 10.1107/S1600536809030554

**Published:** 2009-08-12

**Authors:** Guo-Ying Zhang

**Affiliations:** a College of Chemistry and Life Science, Tianjin Normal University, Tianjin 300074, People’s Republic of China

## Abstract

In the title compound, C_5_H_7_N_3_OS_2_, inversion dimers linked by pairs of N—H⋯N hydrogen bonds occur, forming *R*_2_^2^(8) ring motifs. These dimers are arranged into chains *via* inter­molecular C—H⋯O hydrogen bonds between the methylsulfanyl groups and the O atoms of the carbonyl groups. The acetamido-1,3,4-thio­diazole unit is essentially planar [r.m.s. deviation 0.045 (8) Å].

## Related literature

For the applications of 1,3,4-thio­diazole and its derivatives in anti­microbial drugs and in the construction of metal-organic frameworks, see: Gardinier *et al.* (2007 ▶); Mrozek *et al.* (2000 ▶); Xue *et al.* (2008 ▶). For the synthesis, see: Clerici & Pocar (2001 ▶).
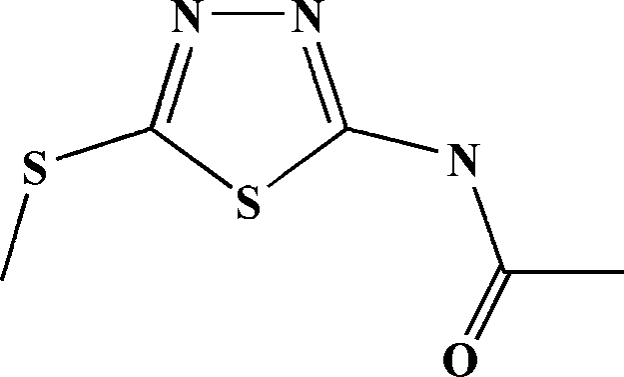


## Experimental

### 

#### Crystal data

C_5_H_7_N_3_OS_2_
*M* *_r_* = 189.26
Triclinic, 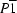

*a* = 5.0797 (10) Å
*b* = 7.9894 (16) Å
*c* = 10.081 (2) Å
α = 91.96 (3)°
β = 90.94 (3)°
γ = 105.27 (3)°
*V* = 394.32 (14) Å^3^
*Z* = 2
Mo *K*α radiation
μ = 0.62 mm^−1^
*T* = 293 K
0.30 × 0.30 × 0.10 mm

#### Data collection

Rigaku R-AXIS RAPID-S diffractometer
Absorption correction: multi-scan (*SADABS*; Bruker, 1998 ▶) *T*_min_ = 0.836, *T*_max_ = 0.941
3437 measured reflections
1382 independent reflections
1259 reflections with *I* > 2σ(*I*)
*R*_int_ = 0.016

#### Refinement

*R*[*F* ^2^ > 2σ(*F* ^2^)] = 0.029
*wR*(*F* ^2^) = 0.078
*S* = 1.07
1382 reflections
106 parameters
H atoms treated by a mixture of independent and constrained refinement
Δρ_max_ = 0.25 e Å^−3^
Δρ_min_ = −0.24 e Å^−3^

### 

Data collection: *CrystalClear* (Rigaku/MSC, 2005 ▶); cell refinement: *CrystalClear*; data reduction: *CrystalClear*; program(s) used to solve structure: *SHELXS97* (Sheldrick, 2008 ▶); program(s) used to refine structure: *SHELXL97* (Sheldrick, 2008 ▶); molecular graphics: *SHELXTL* (Sheldrick, 2008 ▶); software used to prepare material for publication: *SHELXTL*.

## Supplementary Material

Crystal structure: contains datablocks I, global. DOI: 10.1107/S1600536809030554/bq2155sup1.cif

Structure factors: contains datablocks I. DOI: 10.1107/S1600536809030554/bq2155Isup2.hkl

Additional supplementary materials:  crystallographic information; 3D view; checkCIF report

## Figures and Tables

**Table 1:** Hydrogen-bond geometry (Å, °)

*D*—H⋯*A*	*D*—H	H⋯*A*	*D*⋯*A*	*D*—H⋯*A*
N3—H3⋯N2^i^	0.77 (2)	2.12 (2)	2.881 (2)	173 (2)
C1—H1*B*⋯O1^ii^	0.96	2.58	3.289 (3)	131 (2)

Symmetry codes: (i) 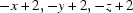; (ii) 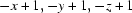.

**Fig. 1.**
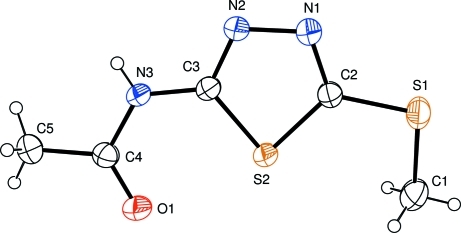
The molecular structure of (I), with the atom-numbering scheme. Displacement ellipsoids are drawn at the 30% probability level.

**Fig. 2.**
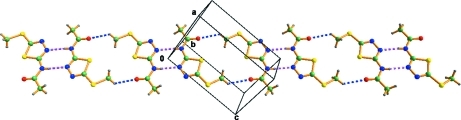
The chain structure linked by N—H···N (pink) and C—H···O (blue) hydrogen bonds in (I).

## supplementary crystallographic information

### Comment

1,3,4-Thiodiazole is important for biological systems, and its derivatives have attracted widespread interest due to their further expanded application in antimicrobial drugs and in the construction of some interesting metal-organic frameworks (Gardinier *et al.*, 2007; Mrozek *et al.*, 2000; Xue *et al.*, 2008). Recently, we synthesized a new thiodiazole-ligand, namely 2-acetamido-5-methylmercapto-1,3,4-thiodiazole, (I). Herein we report the crystal structure of this ligand.

The molecular structure of (I) is shown in Fig. 1. The acetamido-1,3,4-thiodiazole moiety is essentially planar (r.m.s. deviation 0.045 (8) Å), forming a dihedral angle with the C1, S1 and C2 plane of atoms of 14.6 (9)°. In the crystal, inversion dimers linked by pairs of N—H···N hydrogen bonds occur, forming *R*_2_^2^(8) ring motifs. These dimers are arranged into chains *via* intermolecular C—H···O hydrogen bonds between the methyl groups and the O atoms of the carbonyl groups (Fig. 2).

### Experimental

The title compound was prepared according to the literature (Clerici *et al.*, 2001). 5-Methylsulfanyl-1,3,4-thiadiazol-2-ylamine (3.239 g, 0.022 mol) was suspended in acetic anhydride (2.28 ml, 0.024 mol), and acetic acid (9 ml) was added under stirring. The reaction mixture was further stirred at 313 K for 20 min. After cooling, water (10 ml) was added to the mixture, and then the precipitate was recrystallized in EtOH, which gave single crystals suitable for X-ray diffraction analysis (yield: 3.331 g, 80%).

### Refinement

All H atoms bound to C atoms were geometrically positioned and refined using a riding model, with C—H = 0.96 Å and *U*_iso_(H) = *U*_eq_(C). H atom on amino N was located from difference Fourier map and its position was refined freely, with *U*_iso_(H) = *U*_eq_(N). The refined N—H distance is 0.77 (2) Å.

### Crystal data

**Table:**

C_5_H_7_N_3_OS_2_	*Z* = 2
*M**_r_* = 189.26	*F*(000) = 196
Triclinic, *P*1	*D*_x_ = 1.594 Mg m^−^^3^
Hall symbol: -P 1	Mo *K*α radiation, λ = 0.71073 Å
*a* = 5.0797 (10) Å	Cell parameters from 3437 reflections
*b* = 7.9894 (16) Å	θ = 3.3–27.6°
*c* = 10.081 (2) Å	µ = 0.62 mm^−^^1^
α = 91.96 (3)°	*T* = 293 K
β = 90.94 (3)°	Block, yellow
γ = 105.27 (3)°	0.30 × 0.30 × 0.10 mm
*V* = 394.32 (14) Å^3^	

### Data collection

**Table:**

Rigaku R-AXIS RAPID-S diffractometer	1382 independent reflections
Radiation source: fine-focus sealed tube	1259 reflections with *I* > 2σ(*I*)
graphite	*R*_int_ = 0.016
ω scans	θ_max_ = 25.0°, θ_min_ = 3.3°
Absorption correction: multi-scan (*SADABS*; Bruker, 1998)	*h* = −6→6
*T*_min_ = 0.836, *T*_max_ = 0.941	*k* = −9→9
3437 measured reflections	*l* = −11→11

### Refinement

**Table:**

Refinement on *F*^2^	Primary atom site location: structure-invariant direct methods
Least-squares matrix: full	Secondary atom site location: difference Fourier map
*R*[*F*^2^ > 2σ(*F*^2^)] = 0.029	Hydrogen site location: inferred from neighbouring sites
*wR*(*F*^2^) = 0.078	H atoms treated by a mixture of independent and constrained refinement
*S* = 1.07	*w* = 1/[σ^2^(*F*_o_^2^) + (0.0418*P*)^2^ + 0.1675*P*] where *P* = (*F*_o_^2^ + 2*F*_c_^2^)/3
1382 reflections	(Δ/σ)_max_ = 0.001
106 parameters	Δρ_max_ = 0.25 e Å^−^^3^
0 restraints	Δρ_min_ = −0.24 e Å^−^^3^

### Special details

**Table:**

Geometry. All e.s.d.'s (except the e.s.d. in the dihedral angle between two l.s. planes) are estimated using the full covariance matrix. The cell e.s.d.'s are taken into account individually in the estimation of e.s.d.'s in distances, angles and torsion angles; correlations between e.s.d.'s in cell parameters are only used when they are defined by crystal symmetry. An approximate (isotropic) treatment of cell e.s.d.'s is used for estimating e.s.d.'s involving l.s. planes.
Refinement. Refinement of *F*^2^ against ALL reflections. The weighted *R*-factor *wR* and goodness of fit *S* are based on *F*^2^, conventional *R*-factors *R* are based on *F*, with *F* set to zero for negative *F*^2^. The threshold expression of *F*^2^ > σ(*F*^2^) is used only for calculating *R*-factors(gt) *etc*. and is not relevant to the choice of reflections for refinement. *R*-factors based on *F*^2^ are statistically about twice as large as those based on *F*, and *R*- factors based on ALL data will be even larger.

### Fractional atomic coordinates and isotropic or equivalent isotropic displacement parameters (Å^2^)

**Table:**

	*x*	*y*	*z*	*U*_iso_*/*U*_eq_	
C1	0.8654 (4)	0.6728 (3)	0.3920 (2)	0.0434 (5)	
H1A	0.8055	0.5632	0.4333	0.065*	
H1B	0.9004	0.6534	0.3002	0.065*	
H1C	0.7261	0.7336	0.3984	0.065*	
C2	1.0570 (4)	0.8285 (2)	0.63353 (18)	0.0267 (4)	
C3	0.8380 (4)	0.8321 (2)	0.83774 (18)	0.0260 (4)	
C4	0.4332 (4)	0.6973 (2)	0.95823 (19)	0.0282 (4)	
C5	0.2992 (4)	0.7042 (3)	1.0889 (2)	0.0358 (5)	
H5A	0.1062	0.6539	1.0780	0.054*	
H5B	0.3327	0.8229	1.1205	0.054*	
H5C	0.3730	0.6404	1.1521	0.054*	
H3	0.732 (5)	0.878 (3)	1.008 (2)	0.039 (7)*	
N1	1.2055 (3)	0.9415 (2)	0.71786 (16)	0.0324 (4)	
N2	1.0764 (3)	0.9425 (2)	0.83800 (16)	0.0312 (4)	
N3	0.6785 (3)	0.8170 (2)	0.94718 (17)	0.0300 (4)	
O1	0.3383 (3)	0.59336 (19)	0.86769 (14)	0.0406 (4)	
S1	1.17277 (10)	0.80015 (7)	0.47435 (5)	0.03824 (18)	
S2	0.74395 (9)	0.71212 (6)	0.69103 (5)	0.02978 (17)	

### Atomic displacement parameters (Å^2^)

**Table:**

	*U*^11^	*U*^22^	*U*^33^	*U*^12^	*U*^13^	*U*^23^
C1	0.0426 (13)	0.0547 (14)	0.0290 (11)	0.0073 (11)	−0.0009 (9)	−0.0095 (10)
C2	0.0247 (9)	0.0298 (10)	0.0247 (9)	0.0057 (7)	0.0016 (7)	0.0004 (7)
C3	0.0256 (10)	0.0268 (9)	0.0237 (10)	0.0041 (8)	0.0004 (7)	−0.0039 (7)
C4	0.0253 (10)	0.0280 (10)	0.0298 (10)	0.0046 (8)	0.0011 (8)	−0.0005 (8)
C5	0.0328 (11)	0.0379 (11)	0.0336 (11)	0.0036 (9)	0.0085 (9)	0.0009 (9)
N1	0.0286 (9)	0.0358 (9)	0.0290 (9)	0.0021 (7)	0.0051 (7)	−0.0041 (7)
N2	0.0264 (9)	0.0336 (9)	0.0279 (9)	−0.0012 (7)	0.0038 (7)	−0.0062 (7)
N3	0.0266 (9)	0.0327 (9)	0.0246 (9)	−0.0017 (7)	0.0023 (7)	−0.0087 (7)
O1	0.0339 (8)	0.0409 (8)	0.0369 (8)	−0.0064 (6)	0.0029 (6)	−0.0117 (7)
S1	0.0323 (3)	0.0532 (4)	0.0271 (3)	0.0080 (2)	0.0066 (2)	−0.0043 (2)
S2	0.0256 (3)	0.0336 (3)	0.0247 (3)	−0.0007 (2)	0.00124 (19)	−0.00701 (19)

### Geometric parameters (Å, °)

**Table:**

C1—S1	1.796 (2)	C3—S2	1.7244 (19)
C1—H1A	0.9600	C4—O1	1.216 (2)
C1—H1B	0.9600	C4—N3	1.365 (3)
C1—H1C	0.9600	C4—C5	1.499 (3)
C2—N1	1.294 (3)	C5—H5A	0.9600
C2—S2	1.737 (2)	C5—H5B	0.9600
C2—S1	1.7457 (19)	C5—H5C	0.9600
C3—N2	1.297 (2)	N1—N2	1.387 (2)
C3—N3	1.369 (3)	N3—H3	0.77 (2)
			
S1—C1—H1A	109.5	N3—C4—C5	114.83 (17)
S1—C1—H1B	109.5	C4—C5—H5A	109.5
H1A—C1—H1B	109.5	C4—C5—H5B	109.5
S1—C1—H1C	109.5	H5A—C5—H5B	109.5
H1A—C1—H1C	109.5	C4—C5—H5C	109.5
H1B—C1—H1C	109.5	H5A—C5—H5C	109.5
N1—C2—S2	115.25 (14)	H5B—C5—H5C	109.5
N1—C2—S1	120.67 (15)	C2—N1—N2	111.35 (16)
S2—C2—S1	124.08 (11)	C3—N2—N1	112.70 (15)
N2—C3—N3	120.95 (17)	C4—N3—C3	124.71 (17)
N2—C3—S2	114.78 (14)	C4—N3—H3	117.4 (18)
N3—C3—S2	124.27 (14)	C3—N3—H3	117.8 (18)
O1—C4—N3	121.00 (18)	C2—S1—C1	101.30 (10)
O1—C4—C5	124.16 (18)	C3—S2—C2	85.91 (9)
			
S2—C2—N1—N2	0.3 (2)	S2—C3—N3—C4	4.8 (3)
S1—C2—N1—N2	−179.74 (13)	N1—C2—S1—C1	−166.91 (17)
N3—C3—N2—N1	−178.87 (17)	S2—C2—S1—C1	13.04 (15)
S2—C3—N2—N1	0.6 (2)	N2—C3—S2—C2	−0.35 (15)
C2—N1—N2—C3	−0.6 (2)	N3—C3—S2—C2	179.09 (17)
O1—C4—N3—C3	−0.2 (3)	N1—C2—S2—C3	0.01 (16)
C5—C4—N3—C3	178.85 (18)	S1—C2—S2—C3	−179.95 (13)
N2—C3—N3—C4	−175.77 (18)		

### Hydrogen-bond geometry (Å, °)

**Table:**

*D*—H···*A*	*D*—H	H···*A*	*D*···*A*	*D*—H···*A*
N3—H3···N2^i^	0.77 (2)	2.12 (2)	2.881 (2)	173 (2)
C1—H1B···O1^ii^	0.96	2.58	3.289 (3)	131 (2)

Symmetry codes: (i) −*x*+2, −*y*+2, −*z*+2; (ii) −*x*+1, −*y*+1, −*z*+1.
